# Immunohistochemical Characterization of Immune Infiltrate in Tumor Microenvironment of Glioblastoma

**DOI:** 10.3390/jpm10030112

**Published:** 2020-09-03

**Authors:** Hassan Rahimi Koshkaki, Simone Minasi, Alessio Ugolini, Gianluca Trevisi, Chiara Napoletano, Ilaria G. Zizzari, Marco Gessi, Felice Giangaspero, Annunziato Mangiola, Marianna Nuti, Francesca R. Buttarelli, Aurelia Rughetti

**Affiliations:** 1 Department of Experimental Medicine, “Sapienza” University of Rome, Viale Regina Elena, 324-00161 Rome, Italy; hassan.rahimi@uniroma1.it (H.R.K.); alessiougolini217@gmail.com (A.U.); chiara.napoletano@uniroma1.it (C.N.); ilaria.zizzari@uniroma1.it (I.G.Z.); marianna.nuti@uniroma1.it (M.N.); 2 Department of Radiological, Oncological and Anatomo-Pathological Sciences, “Sapienza” University of Rome, Viale Regina Elena, 324-00161 Rome, Italy; simone.minasi@uniroma1.it (S.M.); felice.giangaspero@uniroma1.it (F.G.); francesca.buttarelli@uniroma1.it (F.R.B.); 3 Neurosurgical Unit, Ospedale Santo Spirito, Via Fonte Romana, 8-65124 Pescara, Italy; gianluca.trevisi@ausl.pe.it (G.T.); annunziato.mangiola@ausl.pe.it (A.M.); 4 Neuropathology Unit, Department of Pathology Fondazione Policlinico Universitario “A. Gemelli” IRCCS, Università Cattolica S.Cuore, 00168 Roma, Italy; marco.gessi@policlinicogemelli.it; 5 IRCCS Neuromed, 86077 Pozzilli, Italy; 6 Department of Neurosciences, Imaging and Clinical Sciences, “G. d’Annunzio” University, via dei Vestini, 32-66013 Chieti, Italy

**Keywords:** glioblastoma, immune infiltrate, immune suppression, microenvironment, immune checkpoint, PD-1, PDL-1, IDO, TIGIT, CD163

## Abstract

Background: Glioblastoma (GBM) is the most common primary malignant brain cancer in adults, with very limited therapeutic options. It is characterized by a severe immunosuppressive milieu mostly triggered by suppressive CD163^+^ tumor-associated macrophages (TAMs). The efficacy of immune checkpoint inhibitor interventions aimed at rescuing anti-tumor immunity has not been proved to date. Thus, it is critically important to investigate the immunomodulatory mechanisms acting within the GBM microenvironment for the better design of immunotherapeutic strategies. Methods: The immunohistochemical analysis of a panel of immune biomarkers (CD3, FoxP3, CD163, IDO, PDL-1, PD-1 and TIGIT) was performed in paired samples of the tumor core (TC) and peritumoral area (PTA) of nine GBM patients. Results: CD163^+^ cells were the most common cell type in both the PTA and TC. IDO and PDL-1 were expressed in most of the TC samples, frequently accompanied by TIGIT expression; on the contrary, they were almost absent in the PTA. CD3^+^ cells were present in both the TC and PTA, to a lesser extent than CD163^+^ cells; they often were accompanied by PD-1 expression, especially in the TC. FoxP3 was scarcely present. Conclusion: Distinct inhibitory mechanisms can act simultaneously in both the TC and PTA to contribute to the strong immunosuppression observed within the GBM microenvironment. Nevertheless, the PTA shows strongly reduced immunosuppression when compared to the TC, thus representing a potential target for immunotherapies. Moreover, our results support the working hypothesis that immunosuppression and T-cell exhaustion can be simultaneously targeted to rescue anti-tumor immunity in GBM patients.

## 1. Introduction

Glioblastoma (GBM, Grade IV WHO) is the most common and deadly primary brain tumor, with a median overall survival (OS) of less than 15 months, despite aggressive treatment. High genomic instability, infiltrative behavior and marked angiogenesis are biological traits that confer rapid progression. [1]

A subpopulation of tumorigenic stem-like cells (GBM stem cells) has been suggested as being responsible for the triggering, maintenance and progression of the tumor and appear to be so for GBM resistance to therapies and the high rate of recurrence [2]. Limited therapeutic options are available and, despite new therapeutic strategies having been explored, the prognosis remains extremely poor [3].

The most established molecular biomarker for predicting the outcome of GBM is methylation of the O6-methylguanine-DNA methyltransferase (MGMT) promoter, associated with a positive response to temozolomide, which is used in first-line treatment in combination with radiotherapy [4,5].

On the basis of the presence of recurrent hotspot mutations in isocitrate dehydrogenases (IDH1/2), GBMs are currently distinguished as isocitrate dehydrogenase (IDH)-mutant or IDH-wildtype. IDH mutations are found in the majority of secondary GBMs (70–80%) but only rarely in primary GBMs [6]. Patients with IDH-mutated GBM have evidently better outcomes [7]. Interestingly, this molecular feature has been correlated with a quiescent immunological profile in GBM [8]. Neo-angiogenesis is a characteristic biological trait in GBM development and expansion. The vascular endothelial growth factor (VEGF) produced in response to hypoxic stimuli promotes angiogenesis and vascular permeability. Indeed, targeting VEGF by means of the biological drug Bevacizumab (Avastin^TM^) is currently a standard therapeutic option in second-line treatment [9]. Despite the advances in GBM biology, comprising molecular features and the genetic landscape, only a few targeted therapies—also affecting the VEGF pathway—have been developed and applied [10].

From an immunological point of view, GBM is characterized by a immunosuppressive microenvironment [11]; even though the numbers of T-cells are typically normal among patients, an immunosuppressive population of CD163^+^ M2-like tumor-associated macrophages (M2-like TAMs) tends to be prevalent [12], spurring recent interest in this population as a possible target for novel strategies in immunotherapy. In fact, this population has been largely described as strongly able to suppress T-cell functionality.

The production of the immunosuppressive enzyme indoleamine 2,3-dioxygenase (IDO) as well as of VEGF itself and the expression of co-inhibitory immune checkpoint molecules such as PD-1/PDL-1 are immune mechanisms fostering the immunosuppressive milieu, dampening the T-cell response and thus negatively affecting the outcomes of GBM patients [9,13,14]. Despite the increasing efforts in this field, immunotherapeutic approaches still struggle to achieve good results in GBM patients [15,16,17,18].

The majority of studies on GBM immune infiltrates have historically focused on the bulk of the tumor, but several recent studies have also investigated the peritumoral area (PTA), finding it to be a potential target for novel therapeutic strategies and possible correlations between PTA characteristics and tumor progression and the prognosis of the patients [19,20,21,22,23,24,25,26]. Although this novel aspect has clearly been studied, a general characterization of GBM patients in order to identify the distribution of the principal immunological markers and the molecules targeted by the most common immunotherapeutic strategies, in both the tumor core (TC) and the PTA, remained lacking. In this study, we performed expression analysis by immunohistochemistry of the immune infiltrate, considering a panel of immune checkpoint molecules in the TC and PTA of nine GBM patients.

## 2. Materials and Methods

### 2.1. Patients and Tissue Samples

The patients enrolled in this study underwent surgery for supratentorial, hemispheric GBM at the Neurosurgery Department, Policlinico Agostino Gemelli, Catholic University of Rome. In all cases, total tumor removal was achieved—namely, a resection of the entire contrast-enhancing lesion confirmed by gadolinium MRI—providing samples from both the tumor and the surrounding macroscopic brain tissue appearing to be non-neoplastic at microsurgical inspection (1–2 cm from the tumor border; larger resections were performed for tumors that grew far beyond eloquent areas). All patients provided written consent for the use of their specimens for research purposes; none was identifiable. The local ethics committee approved the study (Catholic University Ethics Committee, Rome, n° A/66/CE/2009), and the ethical principles set out by the Declaration of Helsinki were strictly followed.

Formalin-fixed paraffin embedded (FFPE) tissue specimens from nine adult patients were analyzed.

All tumors, including the tumor cores and the matched periphery tissue samples, were evaluated by two neuropathologists according to the World Health Organization (WHO) classification of tumors of the central nervous system.

### 2.2. Immunohistochemistry

Immunohistochemistry (IHC) was performed by an automatic or manual procedure, via the streptavidin-biotin-immunoperoxidase technique on serial and consecutive 3 µm sections from FFPE blocks. Supplementary Table S1 reports the antibodies and details of the IHC experimental procedure.

Automatic IHC was performed on an automated immunohistochemical stainer (Leica Bond III) with specific antibodies (Ab) against GFAP, R132H mutation in isocitrate dehydrogenase 1 (IDH1-R132H), vascular endothelial growth factor (VEGF), CD3, programmed death 1 (PD-1) and PD-1 ligand (PDL-1), diluted with BOND Primary Antibody Diluent (Leica Biosystems, Newcastle, United Kingdom) according to the manufacturer’s instructions. The BOND Polymer Refine Detection system (Leica Biosystems), supplied ready-to-use for the automated BOND system, was employed.

For manual IHC staining, tissue slides were deparaffinized in xylene, rehydrated through a graded series of ethanol dilutions and then incubated for 15 min in 3% H_2_O_2_ in methanol to inhibit endogenous peroxidases. Antigen retrieval was performed by microwave treatment in 1 x sodium citrate buffer (pH 6.0) for 5 min, three times.

The sections were then washed in phosphate-buffered saline (PBS) and Tween 20 (3 min, 0.1% Tween 20 in PBS; 3 min PBS), and each diluted primary Ab was added to the tissue; the mixtures were incubated overnight at 4 °C. The following Abs were employed: anti-Foxp3, CD163, anti-IDO and anti-TIGIT (see Supplementary Table S1 for details). For IDO and TIGIT, the blocking of non-specific binding was performed for 5 min using protein-blocking buffer (ScyTek Laboratories, Logan, UT, USA). Particular CD163 and IDO expression was consistently measured on consecutive serial sections of PTA or TC samples.

For detection, the UltraTek HRP Anti-Polyvalent (DAB) Staining System/UltraTek Anti-Mouse (AEC) Staining System (ScyTek Laboratories) were used for 15 min at room temperature, according to the manufacturer’s instructions. Diaminobenzidine (DAB) or 3-amino-9-ethylcarbazole (AEC) was used for color development (UltraTek Kit, ScyTek), followed by counterstaining with hematoxylin.

For negative controls, the primary Ab was replaced by normal mouse serum (Sigma). Human normal tonsil was used as a positive control; non-neoplastic tissue obtained from epilepsy surgery was used as a control to compare each Ab stain with the GBM samples.

### 2.3. Evaluation of Immunohistochemical Staining

The number of positive cells was determined independently by two investigators (S.M. and FR.B.) and an experienced neuropathologist (F.G.) blinded to the clinical background of the patients; excellent intra-observer and inter-observer agreement was achieved. The tissue distribution and intensity of each Ab staining was recorded to evaluate biomarker positivity. Vascular, perivascular and infiltrating positive cells were classified. The sections were screened at 20× magnification; the evaluation of positive cells was performed at 40×. Immune cells with apparent morphological appearance different from that of T-cells (for CD3 and FoxP3) or macrophages (for CD163) were excluded from the counts.

### 2.4. ImageJ Analysis of IHC Images

Each slide was digitalized using an Olympus BX51 microscope (Olympus Optical Co., Tokyo, Japan) connected to a digital color camera (Q-Color 5, Olympus) and saved as a jpg file. To compare multiple specimens, staining and image acquisition were performed in parallel for the entire set, using identical settings and exposure times [27].

The image analysis software ImageJ was used to quantify the biomarkers’ expression. ImageJ (NIH, Bethesda MD, USA) is developed and provided for free by the National Institutes of Health (http://rsbweb.nih.gov/ij/); it has specific built-in features including an algorithm to evaluate staining using hematoxylin and diaminobenzidine (DAB) named “color deconvolution H-DAB” [28,29] and has been used for semi-quantitative immunohistochemistry analysis in other systems [28,29,30,31].

Each digitalized image was adjusted by subtracting the background and then processed with “Color deconvolution H-DAB”. The DAB Color 2 images were selected for each biomarker and processed with the thresholding tool [27]. After selecting the threshold, the brown image is converted to a black and white image. The auto setting can be selected or the sliders can be manually moved until all the stained areas are selected, and a histogram is displayed for assistance. The minimum and maximum thresholds were adjusted for each image in order to completely remove the background, without removing the true DAB signal, and then the command “Set” was selected to set the threshold.

The “Mean Grey Value” and “Area Fraction” were subsequently calculated, representing the average intensity of IHC image pixels and the positive area, respectively. The “Mean Grey Value” (*i*) obtained from the software for each image, ranging from 0 (deep brown = highest expression) to 255 (total white = negative), was used to calculate the final DAB intensity (*f*), according to the formula *f* = 255—*i* [29]. Three pictures from the main representative areas were used for each slide; the mean *f* score and the mean “Area Fraction” were calculated.

The “Final IHC Value” was calculated for each sample according to the following formula: Final IHC Value = mean *f* x mean Area Fraction. This takes into account the percentage stained area and mean intensity of the staining, producing a final score representative of IHC positivity.

To limit the intra- and interobserver variability, analyses were performed using the same digital pictures by two independent researchers (S.M. and F.R.B.).

### 2.5. Statistical Analysis

Statistics were determined using GraphPad Prism, version 6 (GraphPad Software Inc., San Diego, CA, USA). The results are expressed as mean values ± SDs. *p*-values were calculated using Student’s *t*-test when comparing two groups of continuous variables. The significance level was defined as *p*-values <0.05 (* *p* < 0.05).

## 3. Results

### 3.1. Patient Characteristics

Different tissue samples were obtained from both the GBM tumor core (TC) and peritumoral area (PTA,) and an immunohistochemical analysis was performed. Table 1 reports patients’ main clinical and anatomo-pathological characteristics. The age range was 30–72 years with a median age of 62.88 years; the female/male ratio was 4/5. This series of cases included six GBMs with frontal localization, two parietal and one temporal cortex. The MGMT methylation status was available for seven patients: Three had unmethylated MGMT. One (GBM07) tested positive for the IDH1 R132H mutation, proving evolution from a previously diagnosed anaplastic astrocytoma. VEGF was analyzed in seven cases, and all tested positive, with diffuse staining. When performed, Ki67 staining was 40–60%. Following surgery, eight patients underwent the Stupp protocol (radiotherapy + temozolomide), while patient GBM02 underwent a palliative treatment with temozolomide alone due to a poor Karnofsky performance status.

### 3.2. Distinct Tissue Localization of Immune Cells in Peritumoral Area vs. Tumor Core

This study analyzed the immune infiltrate in the GBM tissue sections of nine GBM patients, comparing the different immune features of the peritumoral area (PTA) versus the tumor core (TC).

Histopathologically, the periphery of the tumors was defined as the transitional border zone between the normal zone and TC, the latter characterized by a large cellularity area, abundant mitotic figures, widespread necrosis and numerous newly formed vessels. The periphery of the tumors was composed of mixed neoplastic and normal cells; GFAP staining could possibly show the presence of infiltrating neoplastic cells; both the PTA and the TC were compared to the normal brain zone (Supplementary Figure S1). The immune infiltrate was characterized by the presence of CD3^+^ cells (T-cells) and CD163^+^ cells (M2-like tumor associated macrophages, M2-TAMs), regarded as immunosuppressive and pro-tumoral cells [32], and FoxP3^+^ cells (activated T regulatory cells, Treg) in serial and consecutive sections. Furthermore, the immunosuppressive networks were characterized by the expression of the immune checkpoint molecules PD-1, PDL-1 and TIGIT and the enzyme IDO.

**Figure 1:**
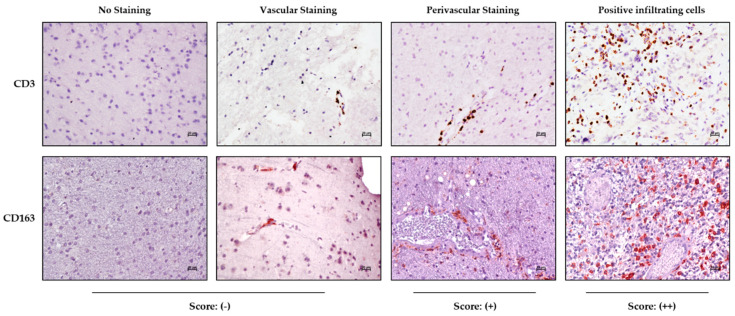
Localization of immune cells in periphery area and tumor core. Representative cases with negative/vascular staining (−), perivascular positivity (+) or infiltrating cells (++) in the parenchyma of tissue for CD3 and CD163 (magnification, 20×).

Immunohistochemical staining in the tumor and periphery was assessed on the basis of tissue distribution as depicted in Figure 1, showing the CD3 and CD163 marker reactivities as an example.

The samples were scored as negative (−) in the absence of reactivity or when positivity was limited to cells inside the vascular lumen; perivascular staining characterized by immune-positive cells having migrated into the perivascular space was indicated as (+), while samples with positive immune cells infiltrating the tumor parenchyma were scored as (++) (Figure 1).

**Figure 2:**
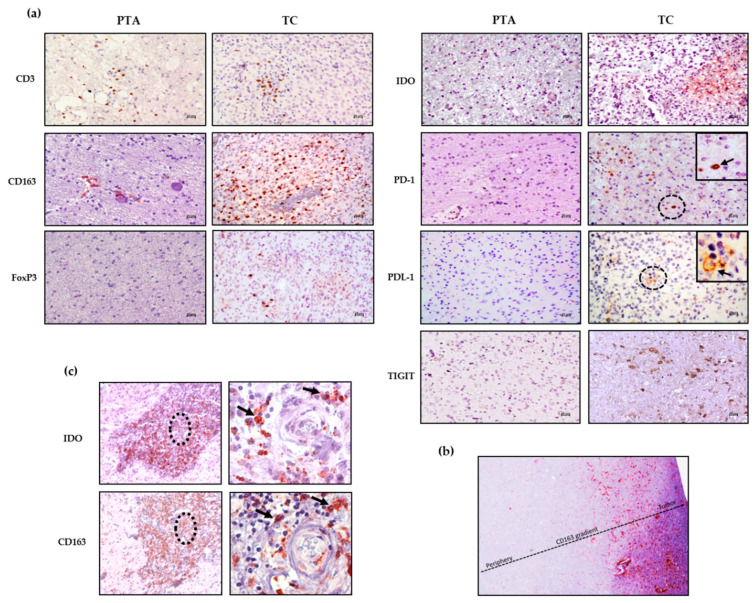
Immune infiltrate distribution in periphery tissue and tumor core of GBM samples. (**a**) Representative expression profile of immune infiltrate as characterized by CD3, FoxP3, PD-1, PDL-1, CD163, IDO and TIGIT in the tumor periphery of GBM (PTA) and tumor core (TC). Magnification, 20× (bar = 20 µm). Detailed images of PD-1- and PDL-1-positive cells in TC are shown (magnification, 40×). (**b**) CD163 positivity gradient from the periphery to the tumor core. CD163^+^ cells increase from the periphery to the tumor core, where they accumulate. The dotted arrow represents the increase in CD163 positive cells from the PTA to TC (magnification, 4×). (**c**) IDO and CD163 expression in GBM05 tumor core sample. Left panels are at 10× magnification; dotted circles identify the perivascular area positive for and IDO and CD163 staining, enlarged in the right panels (40× magnification).

Figure 2a shows representative staining for each marker in the PTA and TC region; while the staining in the PTA is completely negative or positivity associated with the perivascular area, the immune marker expression within the parenchyma is mostly associated with the TC.

Table 2 summarizes the score for each immune marker analyzed in the TC and PTA for each GBM patient. Notably, both the CD3 and CD163 staining in the PTA were positive mainly at the perivascular level, while in the TC, the same staining could be largely found inside the parenchyma, thus indicating a preferential infiltration of CD3^+^ and CD163^+^ cells into the tumor tissue when compared to the periphery, probably due to a strong recruitment exerted by the GBM cells. Immunosuppressive markers such as PD-1, PDL-1, TIGIT, IDO and FoxP3 were found in the TC and not in the PTA. Most of the markers were not detectable in the non-neoplastic brain tissues, where an intravascular positivity regarding CD3 and CD163 could be observed (Supplementary Figure S2).

This observation suggests that the recruitment of immune cells in brain tissue areas that are close to the tumor but not neoplastic may be a consequence of the tumor’s presence, probably triggered by an inflammatory milieu.

Among the different patients, the immunosuppressive population of M2-like TAMs (CD163^+^) is the most represented in both the TC and PTA, showing a type of positivity gradient from the periphery to the core of the tumor (Figure 2b), suggesting a strong recruitment of these cells to the TC. From Figure 2c, it is also possible to observe expression of CD163 and IDO in the same tissue area, reinforcing the immunosuppressive profile of cells such as M2-like TAMs, which not only express PDL-1 but may also release IDO [13], both affecting T effector cell function. This scenario highlights the fact that in the presence of a CD163^+^ population with such a direct negative effect on anti-tumor immune cells, treatment via the activation of effector cells would be difficult without either depleting CD163^+^ cells or reprogramming their immunosuppressive functions.

Moreover, it is possible to note that most of the patients showed a notable presence of immunological markers within the TC, at both the perivascular and infiltrating levels, with a significant prevalence of immunosuppressive markers such as CD163, IDO, PDL-1 and, to a lesser extent, TIGIT and FoxP3. These data confirm the prevalence of a pro-tumoral immune system within the GBM tumor microenvironment.

However, the PTA showed a marked deficit of immune cells, which were mostly localized at the perivascular level. This could suggest a stronger recruitment of immune cells by the tumor preventing their accumulation in the periphery. Notably, previous papers reported that vascular features differed between the PTA and TC, probably leading to the prevalent localization of immune cells in this area [33].

### 3.3. Immune Cells Mediating Suppressive Mechanisms Infiltrate the Tumor Core

**Figure 3:**
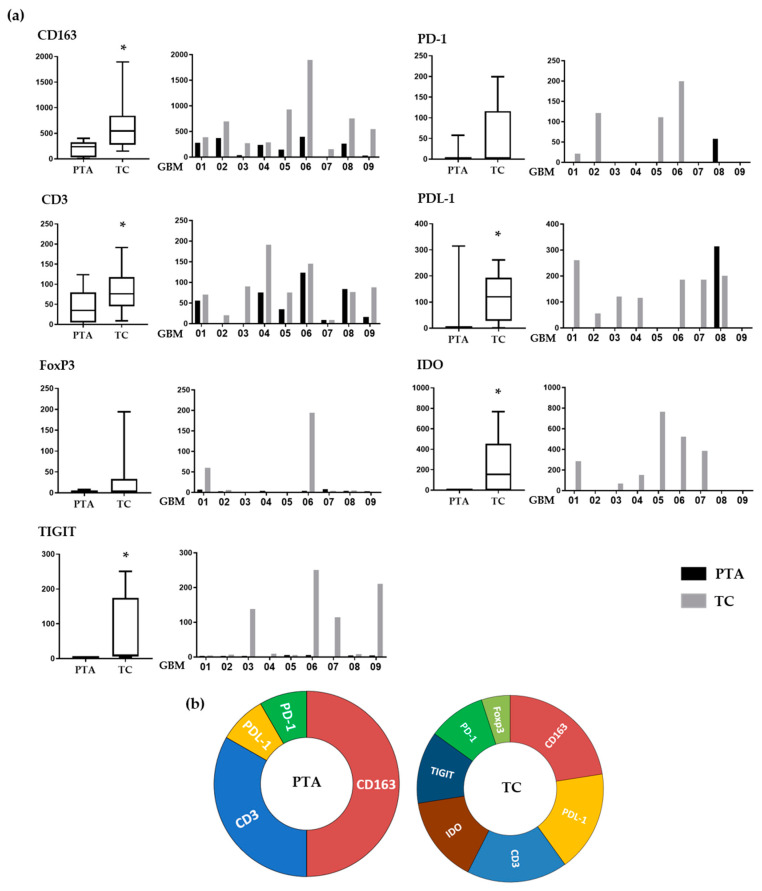
Immune subset distribution in peritumoral area (PTA) and tumor core (TC) of GBM samples. (**a**) IHC values associated with immune markers in the periphery or tumor core depicted for each immune marker. IHC values were obtained by combining the staining intensity and positive area present in the sample tissue area analyzed with the ImageJ software and are reported as arbitrary units (*y* axis); *, *p* < 0.05. (**b**) Immune cell subset infiltrating periphery vs. tumor as depicted by pie charts.

In order to standardize the staining scores, we adapted parameters suggested by the ImageJ analysis software. We assigned to each marker expression a score that combined the intensity and area of positive staining, allowing us to evaluate and compare the expression of each immune marker for each patient in both the periphery and tumor core (Figure 3a).

Regarding CD163, which identifies M2-like macrophages, its positivity was significantly lower in the PTA than in the TC (*p* = 0.02) in each sample tested, showing a gradient that indicates a consistent engagement of these cells by the cancer. This is coherent with their pro-tumoral role, these cells being a source of biological mediators that facilitate tumor growth such as VEGF, PDL-1 and IDO.

The T-cell population, identified through CD3 positivity, was more prevalent in the TC than in the PTA (*p* = 0.02) in every sample, except GBM07 (IDH-mut), who was almost negative for T-cells in both areas. Importantly, these cells showed a similar gradient compared to that of the CD163^+^ cells, suggesting two important things. On one hand, CD163^+^ and CD3^+^ cells may be in close proximity, confirming the suppressive effect of TAMs on T-cells, increasing the interest in targeting these cells to potentiate T-cells’ effector functions. Additionally, T-cells are recruited at the tumor site and could potentially be targeted by immunotherapeutic drugs. The fact that T-cells encounter a strong immunosuppressive microenvironment once reaching the TC may account for the failure of all of the recent trials of immune checkpoint inhibitors [34,35,36].

FoxP3 positivity, indicating the presence of Treg cells, was found only in the TC of GBM01 and 06 samples, while all the PTAs tested were negative.

IDO was expressed in most of the samples tested, exclusively within the TC areas (*p* < 0.02). Importantly, IDO positivity localized with CD163 positive cell clusters in most of the samples tested (as also shown in Figure 2c), suggesting that these cells are a source of IDO in GBM. This tremendous accumulation inside the microenvironment could evidence the potential benefit of an immunotherapy based on the targeting of this immunosuppressive enzyme on one hand, while, on the other hand, it further highlights the need to target CD163^+^ cells, which are partly responsible for its production.

The number of PD-1-positive cells, when present, was higher in TC than PTA. Interestingly, the only patient showing PD-1 positivity in the PTA (GBM08) had no PD-1 in the TC. PD-1 is one of the most important exhaustion receptors on T-cells, so this may suggest that T-cells experiencing exhaustion in the PTA are not able to reach the TC. Another interesting observation is that while all the patients tested positive for CD3^+^ cells inside the TC, only four were also positive for PD-1. This could be explained by the fact that T-cells recruited at the tumor site experience a strong suppression exerted by the TAMs, so they are not even able to be properly activated and, consequently, express exhaustion receptors. This lack of PD-1 expression as a possible consequence of TAM immunosuppression inside the TC may partly explain the failure of anti-PD-1 therapies and strengthen the need for blocking the negative effects of TAMs before targeting T-cells.

PDL-1 positivity showed a similar trend, with the majority of the samples showing higher values in the TC, while only GBM08 showed an opposite pattern: no PDL-1 positivity in the TC but some expression in the PTA. It is interesting that the higher positivity for PDL-1 was strictly associated with higher numbers of CD163^+^ cells, thus confirming that M2-like TAMs are the main cells expressing this immunosuppressive ligand. Moreover, 7/9 samples showed the presence of >1% PDL1-positive cells in the tumor core. This is an important evidence of how the PDL-1/PD-1 suppressive pathway is also relevant in the GBM tumor microenvironment; the fact that different trials involving anti-PD-1/PDL-1 drugs have failed should thus be imputed to additional mechanisms involved in effector T-cell impairment.

Regarding TIGIT, a marker expressed on suppressive T-cells [37], positive cells were found in four TC samples (GBM03, 06, 07 and 09) with no expression in the periphery (*p* = 0.02). Its presence in the TC suggests that when T-cells are exposed to such a hypoxic and immunosuppressive environment as the one found inside the GBM, they can be altered to a suppressive phenotype. Indeed, it is important to note in our samples the association of TIGIT expression with other suppressive molecules such as IDO and PDL-1. Figure 3b summarizes the different immune profiles present in the periphery and tumor core, showing how distinct co-inhibitory and immunosuppressive mechanisms simultaneously occur in the TC and not in the PTA.

### 3.4. Distinct Immunosuppressive Mechanisms Can Cooperate in GBM Patients

**Figure 4:**
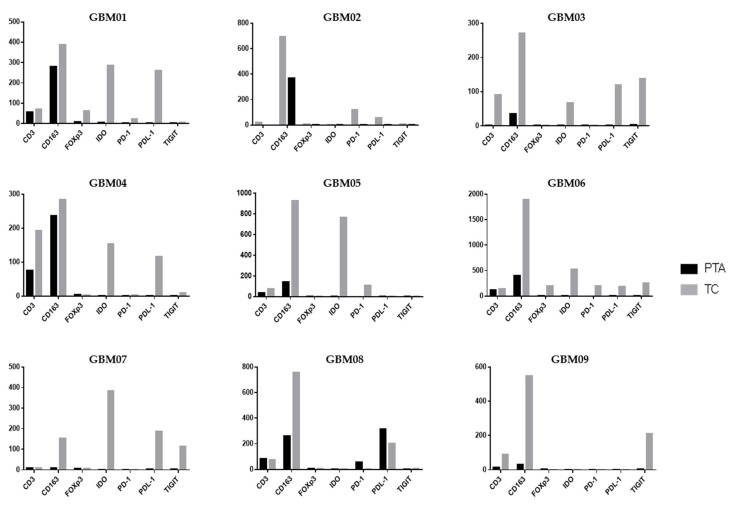
Immune cell profile in each GBM patient sample. IHC values for each immune marker are displayed for each GBM patient sample. Histograms represent the IHC values obtained with the ImageJ software and reported as arbitrary units (*y* axis).

Following the analysis of the single markers, it is also important to contextualize them within the single patient picture, unifying the observations in TC vs. PTA (Figure 4).

In GBM01, the TC showed a highly immunosuppressive TME, with an abundance of cells positive for CD163, IDO and PDL-1 and notable positivity for FoxP3. TIGIT and PD-1 were also slightly positive. However, CD3^+^ cells were rare. In the PTA, the number of CD163^+^ cells was much higher than that of CD3^+^ cells. It is thus unsurprising that the OS of patient GBM01 was only 12 months (Table 1).

GBM03 and GBM04 had a very similar infiltrate distribution pattern to GBM01, with a large abundance of immunosuppressive markers in both the TC and PTA. GBM04 showed a notable presence of T-cells also in the PTA yet associated with a higher number of CD163^+^ cells. These observations suggest that an increase in T-cells in the PTA probably led to the recruitment of many suppressive cells by the tumor to escape immune control. Accompanying the expression of the CD163 marker, we also found co-localization of the IDO immunosuppressive enzyme.

Similarly to GBM03 and 04, GBM09 was characterized by the presence of CD3- and CD163-positive cells in both the PTA and TC, with a higher intensity and number of positive cells in neoplastic areas; TIGIT expression was found in the tumor core, while the periphery was almost completely negative for all the other analyzed markers. As in the other samples, TIGIT was associated with CD163 and PDL-1 expression.

In GBM05, the high expression of IDO was accompanied by a relevant positivity for CD163 in the TC; the positivity for these markers is often co-localized in the cytoplasm of M2-like TAMs in GBM as previously shown in Figure 2 and Figure 3, suggesting the generation of an immunosuppressive macrophage profile induced by neoplastic cells the in tumor core of this GBM. This observation could have suggested an important rationale for the use of an anti-IDO approach in combination with other Immunotherapeutic drugs.

An interesting aspect of the PD-1/PDL-1 expression profile is present in the GBM02 patient who had the shortest OS among the patient cohort. In the TC of this patient, a notable positivity of PD-1 was associated with a high positivity for PDL-1 and CD163 cells. It is important to note that a combination immunotherapeutic approach simultaneously targeting the PD-1/PDL-1 axis and TAMs has been proved efficacious in a mouse model and is a challenging therapeutic option [38]. A peculiar immune profile was found in the GBM08 sample; it was the only one to express PD-1^+^ cells in periphery of the tumor in association with a high expression of PDL-1 and CD163, while no positivity for PD-1 was found in the TC. This co-expression suggests the possibility that tumor cells strictly modulate the expression of immunosuppressive markers in periphery of GBM, activating PDL-1 expression in order to bring T-cells to exhaustion, when present.

The GBM06 and GBM07 samples depict two opposite and extreme patterns of immune infiltrating cells. All the immunosuppressive biomarkers were highly co-expressed in GBM06 in the TC as shown by the histograms; this patient thus showed a hot tumor, enriched for immune cells, that would have rendered them a potential candidate to benefit from an immunotherapeutic approach.

On the other hand, the GBM07 patient showed the lowest positivity for almost all of the biomarkers analyzed in the TC and a total absence of these markers in the PTA. Interestingly, this sample was the only one to harbor the IDH1 mutation, which has been previously shown to be associated with an immune-quiescence of tumors [8].

Taken together, all these data indicate that the immunosuppressive profile is prevalent in both the TC and PTA of GBM, with the periphery showing a marked reduction of immune biomarkers. In addition, the notable presence of TIGIT and its association with other immunosuppressive markers such as CD163 and PDL-1 suggest its possible role as a novel biomarker of an immunosuppressive tumor microenvironment in GBM.

## 4. Discussion

In this study, we proposed the cumulative analysis of a panel of immune biomarkers in order to investigate the immune infiltrate profiles in paired periphery and tumor tissue samples of nine GBM patients. In particular, we investigated the CD3 marker, which identifies anti-tumor immune populations, and CD163 and FoxP3, which indicate myeloid and T-cell immunosuppressive cell subsets, respectively. Moreover, we analyzed some well-established targets for potential immunotherapies, such as the immune checkpoints PD-1, PDL-1, TIGIT and IDO, and investigated their distribution in both the TC and the PTA. In fact, a large number of both the immunotherapies that have already proven their validity and the novel therapies that are currently under investigation for the treatment of GBM patients have the immune system as a direct or indirect target, as in the cases of bevacizumab and regorafenib, respectively [39,40]. Indeed, the targeting of angiogenesis by anti-VEGF antibody as well as TKI inhibitors has shown to impact not only vascularization architecture and permeability but also immune tumor texture as in other tumors [41,42].

In this scenario, knowledge of the localization and distribution of the main immunological markers and targets for the immunotherapies in both the TC and the PTA assumes a primary role. Indeed, in recent years, the study of the PTA in GBM has garnered particular interest, since 90% of recurrence occurs in this area [24]. Notably, several recent studies have focused on the characterization of this peritumoral area in GBM to more deeply elucidate its relevance in GBM progression and find potential therapeutic targets [43]. However, an extended analysis of the expression of the molecules targeted by the immunotherapies in this area is lacking, and it has never been compared with that in the bulk of the tumor to a major extent. Although a small cohort of patients was considered in this study, the results provided interesting information on the profile of the immune infiltrate pattern in PTA vs. TC areas. Our results confirmed that both the GBM microenvironment and its periphery were strongly immunosuppressive. The M2-like TAM population identified by as CD163^+^ cells was the most represented in all the samples tested, with an established superiority when compared to the T-cell population. Furthermore, distinct mechanisms of immunosuppression appeared to be associated with CD163^+^ cells in the TC area. IDO was present in almost all of the TC samples tested and none of the PTA samples. It is interesting to note that VEGF, besides its angiogenic role, stimulates pro-tumoral immunity, triggering distinct immunosuppressive mechanisms [33,44]. We and others have already shown that VEGF expression is lower in the PTA compared to that in the TC. Additionally, the expression of VEGF receptor (VEGFR)-1/2 is either low or absent in the PTA [25,45].

This evidence strongly suggests that the presence of a constant immunosuppressive infiltrate in GBM should be considered when establishing new immunotherapeutic strategies, since the majority of clinical trials involving immune checkpoint inhibitors have not proven their clinical efficacy [15,18,34,35,36,46]

Vaccination with neoantigen-specific T-cells in GBM patients is able to bring these therapeutic activated T-cells to the tumor site, but they end up being unable to induce a clinically relevant response, most probably because of the presence of this strongly suppressive microenvironment that they encounter [16,17]. It is important to note that we demonstrated how both CD163^+^ and CD3^+^ cells showed a similar gradient from the periphery to the tumor area, with most of them localizing in the bulk of the tumor. This similar behavior strongly suggests a close proximity of these two populations, highlighting the immune modulation exerted by TAMs on T-cells, preventing the exploitation of their effector functions that should be expected from T-cell-targeting drugs. The fact that all the patients tested were positive for the CD3 marker in the TC but only some of them expressed the exhaustion receptor PD-1 (expressed on activated T-cells) may further suggest that the consistent suppression that T-cells experience inside the tumor area limits their proper activation [47]. This lack of activation and, consequently, of PD-1 expression inside the tumor may partially explain the failure of T-cell-targeting strategies, raising interest in novel approaches that could improve the effectiveness of classic immunotherapeutic approaches by removing the negative constraints that the GBM TME exerts on T-cells.

Several recent reports characterize the severe exhaustion signature of T-cells in glioblastoma [48] and functional hyporesponsiveness [49]. It has been proposed that distinct co-inhibitory mechanisms are involved; besides the PD-L1/PD-1 axis, LAG3 and, more recently, TIGIT have been detected in glioblastoma T-cells [50,51].

TIGIT is an important marker expressed by suppressive T-cells as demonstrated in other cancer settings [37]. In our study, we outlined that it is expressed in the TC of some patients together with, and to a similar extent as, other well-established immunosuppressive markers, suggesting that when TILs are exposed to a strongly immune-regulative environment as the GBM TME, they can be switched to a pro-tumoral phenotype. In this scenario, our data also suggest that TIGIT could be used as an additional marker of an immunosuppressive microenvironment in GBM and, above all, that can be regarded as potential target for novel strategies in immunotherapy.

Our results indicate a marked reduction in immunosuppressive markers in the periphery when compared to the TC. Interestingly, it has been reported that the PTA hosts more effector T-cells that have not experienced exhaustion than the TC [33]. This would suggest a more suitable environment for the immunotherapies to be effective.

To strengthen these ideas, we reported that the only patient that tested positive for the IDH1 mutation showed a lack of immune cells in both the PTA and TC. It has already been demonstrated that IDH1 is associated with an immune quiescent profile [8]. In our patient cohort, in accordance with previous findings [7], the IDH1-mut patient had a much higher OS. This is strong evidence of how the immune system in the GBM is robustly activated to be suppressive; indeed, its lack, as in the case of the IDH1-mut patient, leads to a better prognosis and better control of the tumor’s growth.

Thus, limiting the immune suppression in combination with an immune stimulation in GBM patients could be a fundamental step in activating an efficient anti-tumor immune response overcoming the limitations that immune-based strategies have encountered to date in clinical trials.

However, each patient carries a “personal” combinatorial immune profile, for which it is crucial to define the location, distribution and association of immune cells within the tumor. This represents not just a picture but an effective tangle between the immune system and cancer cells [52], resulting in distinct biological implications and possibly clinical impact for each patient.

The main limitation of the present study was the small number of samples analyzed, which might not depict the real picture of glioblastoma, limiting the generalizability of our results. Acquiring extensive data on peripheral tissue is of utmost importance, as it represents the actual target of post-operative therapies. Nonetheless, the surgical sampling of brain tissue beyond the radiologically and microsurgically defined tumor border is only possible in a minority of glioblastoma cases due to the risk of adjunctive, unjustified functional damage. Despite these limitations in PTA tissue availability, studies with larger numbers of samples are needed to confirm our findings and better delineate the characteristics of this heterogeneous tissue. Furthermore, such information needs to be interpolated with all the molecular, biological and clinical data available for each single patient in a network medicine-based approach to determine optimal patient-tailored immunotherapeutic strategies.

## 5. Conclusions

Our analysis confirmed the strong heterogeneity that occurs among different patients regarding the “quality” of the immune infiltrate and different mechanisms of immunosuppression that prevail inside diverse tumors [12,24]. Therefore, a detailed immunohistochemical analysis of GBM samples of tumor and periphery tissue could reveal single-patient characteristics along with the prevalent mechanism of suppression and thus aid clinicians in the selection of the best therapeutic plan to achieve the best patient response.

Additional studies with larger cohorts of patients integrating molecular, biological and immune information will be needed in the future to better establish the validity of these speculations.

## Figures and Tables

**Table 1:** GBM patient characteristics.

	Sex	Age	Localization	IDH1 R132H	Ki67	VEGF	MGMTp-meth	Therapy	OS	PFS
**GBM01**	F	64	Frontal sx	Neg	50%	Pos	Un-meth	RT-TMZ (6 cycles TMZ)	12	9
**GBM02**	M	67	Frontal sx	Neg	n.a.	n.a.	n.a.	TMZ palliative	3	3
**GBM03**	M	66	Temporal sx	Neg	60%	Pos	Un-meth	RT-TMZ (4 cycles TMZ)	7	5
**GBM04**	F	66	Frontal dx	Neg	50%	Pos	Meth	RT-TMZ (12 cycles TMZ)	21	17
**GBM05**	F	65	Frontal dx	Neg	n.a.	n.a.	n.a.	RT-TMZ (11 cycles TMZ)	19	16
**GBM06**	M	71	Frontal sx	Neg	40%	Pos	Un-meth	RT-TMZ (4 cycles TMZ)	22	6
**GBM07**	F	30	Parietal dx	Pos	40%	Pos	Meth	RT-TMZ (4 cycles TMZ)	40 alive	6
**GBM08**	M	72	Parietal sx	Neg	40%	Pos	Meth	RT-TMZ (6 cycles TMZ)	n.a.	19
**GBM09**	M	65	Frontal dx	Neg	n.a.	Pos	Meth	RT-TMZ (4 cycles TMZ)	n.a.	n.a.

TMZ: temozolomide; RT: Radiotherapy; n.a.: not available; OS: Overall Survival; PFS: Progression Free Survival. OS and PFS are expressed in months.

**Table 2:** Immune cell distribution in periphery and tumor core.

	CD3		FoxP3		CD163		IDO		PD-1		PDL-1		TIGIT	
	PTA	TC	PTA	TC	PTA	TC	PTA	TC	PTA	TC	PTA	TC	PTA	TC
**GBM01**	+	++	-	++	+	++	-	++	-	++	-	++	-	-
**GBM02**	-	-	-	-	+	++	-	-	-	++	-	++	-	-
**GBM03**	-	++	-	-	-	++	-	++	-	-	-	++	-	++
**GBM04**	+	++	-	-	+	++	-	++	-	-	-	++	-	-
**GBM05**	+	++	-	-	+	++	-	++	-	++	-	-	-	-
**GBM06**	++	++	-	++	+	++	-	++	-	++	-	++	-	++
**GBM07**	-	-	-	-	-	+	-	++	-	-	-	++	-	++
**GBM08**	++	+	-	-	+	++	-	-	++	-	++	++	-	-
**GBM09**	-	++	-	-	-	++	-	-	-	-	-	-	-	++

(-): negative tissue staining or vascular staining; (+): perivascular positivity; (++): positive infiltrating cells; PTA: Peritumoral Area; TC: Tumor Core.

## Supplementary Materials

The following are available online at https://www.mdpi.com/2075-4426/10/3/112/s1. Supplementary Figure S1: Identification of periphery and tumor core area as characterized by immunohistochemical parameters. Supplementary Figure S2: Immune infiltrate distribution in non-neoplastic brain tissue. Supplementary Table S1: Antibodies employed in the study and experimental procedure for immunohistochemical analysis.
