# Supplementary material for: Immunohistochemical Characterization of Immune Infiltrate in Tumor Microenvironment of Glioblastoma
Source: J Pers Med. 2020 Sep 3;10(3):112. doi: 10.3390/jpm10030112 (PMC7564919; doi:10.3390/jpm10030112)
Supplement: Supplementary file 1 [file jpm-10-00112-s001.zip › Supplementary figure and table jpm-902225/Suppl Table S1 (02-09-2020).docx]

**Supplementary Table S1 –** Antibodies employed in the study and experimental procedure for immunohistochemical analysis.

| **Antigen** | **Antibody** | **Manufacturer** | **Staining**  **System** | **Antigen Retrieval Buffer** | **Dilution**  **Incubation** | **Incubation and Staining System** |
| --- | --- | --- | --- | --- | --- | --- |
| **GFAP** | clone GA-5 | Novocastra | Leica Bond III | Citrate, pH 6  20 min | 1:200  15 min | Bond Polymer Refine Detection (DAB) |
| **IDH1 R132H** | clone H09 | Dianova | Leica Bond III | EDTA, pH 8  40 min | 1:50  30 min | Bond Polymer Refine Detection (DAB) |
| **VEGF** | clone 3F7 | Sigma-Aldrich | Leica Bond III | Citrate, pH 6  30 min | 1:100  30 min | Bond Polymer Refine Detection (DAB) |
| **CD3** | clone LN10 | Leica | Leica Bond III | Citrate, pH 6  30 min | 1:50  30 min | Bond Polymer Refine Detection (DAB) |
| **Foxp3** | clone 236A/E7 | Abcam | Manual | Citrate, pH 6 | 1:100  1 hr | UltraTek HRP Anti-Polyvalent (DAB) |
| **CD163** | clone 10D6 | Novus Biologicals | Manual | Citrate, pH 6 | 1:300 | UltraTek Anti-Mouse (AEC) |
| **PD1** | clone NAT105 | Abcam | Leica Bond III | EDTA, pH 8  20 min | 1:50  30 min | Bond Polymer Refine Detection (DAB) |
| **PDL-1** | clone 22C3 | Dako | Leica Bond III | EDTA, pH 8  20 min | 1:50  30 min | Bond Polymer Refine Detection (DAB) |
| **IDO** | clone 10.1 | Millipore | Manual | Citrate, pH 6 | 1:50  overnight | UltraTek  Anti-Mouse (AEC) |
| **TIGIT** | clone TG1 | Dianova | Manual | Citrate, pH 6 | 1:100  overnight | UltraTek HRP Anti-Polyvalent (DAB) |
